# Benchmarking adhesive performance in bonding FDM TPU to PBF PA12 3D-printed parts

**DOI:** 10.1038/s41598-025-32559-w

**Published:** 2025-12-19

**Authors:** Sachintha Alwis Weerasinghe, Adam Kłodowski, R Scott Semken, Grzegorz Orzechowski, Aki Mikkola

**Affiliations:** School of Energy Systems, Lappeenranta-Lahti University of Technology, Lappeenranta, Finland

**Keywords:** Shear, Peel, Tensile, Testing, PA12, TPU, Engineering, Materials science

## Abstract

Additive manufacturing provides an efficient method to rapidly develop prototypes utilizing TPU, which is flexible, and Nylon (PA12), which is known for strength and chemical resistance. Five structural adhesives to bond, without plasma/flame pretreatments, flexible FDM TPU to rigid PBF PA12 were benchmarked using the ASTM D903 (180° peel), D2095 (butt-joint tensile), and D3163/D3164 (lap-shear) standards. The adhesives tested were Loctite HY4070, Permatex Plastic Welder, Mannol 9904, Loctite 401 with 770 primer, and 3M DP8010. Across five samples per test, Loctite 401 + 770 achieved the highest mean strengths with low scatter (COV ≤ 15%) in peel (2.6 N/mm), shear (3.0 MPa), and tensile (2.7 MPa) testing. Occasional adherend-side failures in shear suggested joint strengths approaching that of the substrate. The performances of HY4070 and Permatex were acceptable but fell below that of the Loctite 401 + 770. DP8010 and Mannol underperformed, especially in peel and tensile testing. Stress–displacement curves and failure-surface images suggest these adhesives predominantly experienced cohesive failures. Adhesive TPU-PA12 bonding using Loctite 401 with 770 primer is a robust, simple, and fast assembly approach when the presence of thin bond lines is acceptable. We minimize variability through defined process controls and, for durability-critical products, recommend next-stage tests: aging, impact, UV, and fatigue.

## Introduction

Additive manufacturing enables rapid, functional polymer parts, but combining dissimilar printed polymers to produce a composite part remains challenging. Among common 3D printing processes, FDM (anisotropic extrudates) and PBF (near-isotropic sintered parts) are frequently used to make parts that must be integrated. These parts are often bonded using adhesives, however systematic standardized benchmarking of off-the-shelf adhesives and bonding processes for TPU (flexible, low-surface-energy) to PA12 (low-energy, hygroscopic, chemically resistant) is largely nonexistent.

Plastic-to-plastic joining methods can be categorized into four broad families: mechanical, welded, adhesive bonded, and solvent cementing^3^. Adhesive bonding spreads loads across a wide continuous joint instead of concentrating stress at screws, clips, or snap-fits. It also allows designers to join thin or dissimilar plastics without adding weight or visible hardware, yielding lighter and cleaner-looking parts. Because of these benefits, this research centers on adhesive bonding. The assumption is that modern adhesives will provide a reliable and flexible bond between extruded TPU and sintered nylon 3D-printed elements. Adhesion is influenced by surface contamination, moisture, temperature, roughness, and adhesive aging. Specific characteristics vary across adhesives and manufacturers.

Common powders used in PBF include PA12 (nylon), thermoplastic polyurethanes or elastomers, polypropylene, and polystyrene. PA12 is particularly popular due to its excellent mechanical properties, dimensional stability, low moisture absorption, good chemical resistance, versatility, and recyclability^1^. PA12, like other polyamides, exhibits a relatively low surface energy of around 40 dyn/cm, which limits its wettability and makes it inherently difficult to bond^2^. In addition, PA12’s hygroscopic nature allows it to absorb up to 3% of its mass in moisture, leading to the formation of weak, water-rich interfacial layers that further undermine adhesive strength^3^. As a result, untreated nylon surfaces typically require surface roughening, energetic pretreatments such as plasma or flame activation, or the use of specialized primers to elevate surface energy and achieve improved surface adhesion.

TPU (thermoplastic polyurethane) is particularly popular as an FDM material due to its superior wear/abrasion resistance, toughness, shock absorption properties, and flexibility, even at low temperatures^4^. TPU makes an excellent grip material. It is a common choice for applications in the automotive, consumer products, medical devices, and fashion industries. TPU is classed as a low-surface-energy material because its surface is dominated by long, non-polar soft-segment chains that offer very few polar or hydrogen-bonding sites. This gives untreated TPU a surface free energy roughly one-third that of metals or many engineering plastics and water contact angles of around 100–110°. As a result, most adhesives or coatings cannot effectively wet TPU^5^.

Adhering TPU to PA12 relies on specific chemical and physical interactions. TPU, characterized by segmented block-copolymer structures with hard (polar urethane) and soft (non-polar polyol) segments, adheres effectively through hydrogen bonding and molecular interdiffusion with the amide groups of PA12. Effective adhesion requires matching surface energies and interfacial contact. Temperature significantly influences adhesion by promoting polymer chain mobility, which facilitates molecular entanglement at interfaces.

This study identified, tested, and ranked readily available adhesives used to bond TPU to PA12 without specialized surface pretreatment. The ASTM D903 (peel), D2095 (tensile), and D3163/D3164 (shear) standards controlled the adhesive bonding processes to prepare five test specimens for each test category. Loctite 401 + 770 was the best performer in peel (2.6 N/mm), shear (3.0 MPa), and tensile (2.7 MPa) testing. The performances of HY4070 and Permatex were acceptable but below that of Loctite 401 + 770. DP8010 and Mannol were the weakest performers.

## Materials and methods

Three separate tests were carried out to determine PA12-to-TPU adhesive bonding performance for five different adhesives. The tests mimicked the three most common failure modes for adhesively bonded joints: peel, tensile, and shear. The test results were analyzed and used to recommend the best performing adhesive for each mode of failure.

### Selection of adhesives for testing

Adhesives were chosen based on availability and online presence. The five adhesives selected for testing were as follows.

1. Loctite Hybrid HY 4070 is a two-part, fast-fixturing, cyanoacrylate/acrylic-hybrid adhesive that combines the strength and gap filling properties of epoxy with the speed of cyanoacrylate^6^.
2. Permatex Plastic Welder is a two-part MMA (methyl-methacrylate) structural adhesive designed specifically for bonding plastics. Permatex Plastic Welder offers gap-filling properties and good impact resistance^7^.
3. Mannol Epoxy-Plastic 9904 is a two-component epoxy adhesive formulated for repairing and bonding plastic components^8^.
4. Loctite 401 + 770 Primer is a combination of fast-bonding ethyl cyanoacrylate adhesive Loctite 401^9^ and a surface-activating primer Loctite 770^10^. It is used for difficult-to-bond materials such as polyolefins (polyethylene and polypropylene).
5. 3M Scotch-Weld DP8010 is a high-performance, two-part MMA structural adhesive designed for bonding, without pre-treatment, low-surface-energy plastics such as polyethylene and polypropylene. The manufacturer also claims the adhesive can be used without surface priming on low surface energy plastics^11^.

### Selection of methods, equipment and specimen size

To determine which adhesives effectively bond the flexible-to-rigid combination of extruded TPU and sintered PA12, ASTM testing regimes were applied that addressed the main ways a joint will be stressed in service.

ASTM methods provide a validated, globally recognized test framework with defined geometries and loading while minimizing variability. Peel strength was tested according to ASTM D903-98^12^, which details a 180-degree peel test. The soft TPU layer was bent back onto itself while the PA12 remained mostly flat. Pure tensile strength of the bond was measured with butt-joint specimens prepared according to ASTM D2095-96^13^ and machined and aligned per ASTM D2094^14^. In-plane shear was covered by two closely related lap-shear standards: ASTM D3163^15^ for relatively rigid plastic adherends and ASTM D3164-03^16^ for sandwiches or slightly flexible parts.

Testing was conducted on a Shimadzu Autograph AGS-X Series Universal Testing Machine (UTM) with a 1 kN load sensor installed.

Five replicates were tested for each adhesive in each of the three test types for a total of 75 mechanical tests (5 specimens × 5 adhesives × 3 methods). Five specimens for each condition were considered sufficient to be 95% sure that strength differences of about 20% are captured because bonded plastic parts normally vary only 10–15% from one specimen to the next.

### Details of the selected testing methods

The purpose of this section is to explain the overall process followed for this set of experiments including adaptation of testing processes based on standards, preparation of the specimens, specimen conditioning, and bonding. All three test specimens were conditioned for specific periods under controlled conditions before testing. Prior to testing, all specimens were allowed to stabilize in the test environment for at least one hour before proceeding with testing to ensure consistency in results.

#### Specimen preparation

For the test reported here, samples were fabricated using generic PBF PA12 and Bambu lab’s shore 95 A HF (High Flow) TPU. The FDM samples were printed in a Bambu lab P1S printer on a 0.4 mm nozzle at a layer height of 0.2 mm, bed temp 35 °C, nozzle temperature of 235 °C, and print speed of 150 mm/s. The samples were printed with the length parallel to the print bed to maximize the strength of the sample. The PBF specimens were manufactured on an EOS P 770. A layer thickness of 0.10–0.12 mm is standard, combined with a chamber temperature around 170 °C to keep the powder just below melting for uniform sintering. The laser spot size of 0.42 mm, with hatch spacing typically 0.25 mm, ensuring dense and well-fused parts.

Specimen surfaces were prepped by lightly sanding using 320 grit sandpaper and blasting the surfaces with high pressure air to remove any sanding debris or particulate matter remaining from the PBF print process. Next, the specimens were placed in an industrial grade Easy Composites Ltd. OV301 curing oven at 50 °C for 24 h to eliminate any moisture absorbed by the PA12, which will reduce adherence^3^. Prior to bonding, the surfaces were wiped clean with isopropanol to provide a residue-free surface.

#### Adhering specimens and using jigs

The application of adhesives was straightforward with the HY4070 and DP8010, which comes with self-mixing nozzles that allow for perfect mixing of the two components that make up each adhesive system. The plastic welder and plastic epoxy are 2-component adhesives that must be mixed manually. For these adhesives, 10 cm x 10 cm pieces of air dusted plywood and wooden skewers were used to mix the components to the desired consistency. Application of Loctite 401 also proved easy. First, the 770 primer was brushed onto each surface. After letting it sit for 1 min, the 401 adhesive was applied.

Jigs and plates were used to ensure consistent pressure and alignment of the bonded specimens. Two sheets of 15 mm birch plywood were used with 4 clamps for the shear and peel specimens. For the tensile specimens, a jig was designed that simultaneously provided alignment of the cylindrical faces and parallel alignment of the holes for the locking pins.

**Fig. 1:**
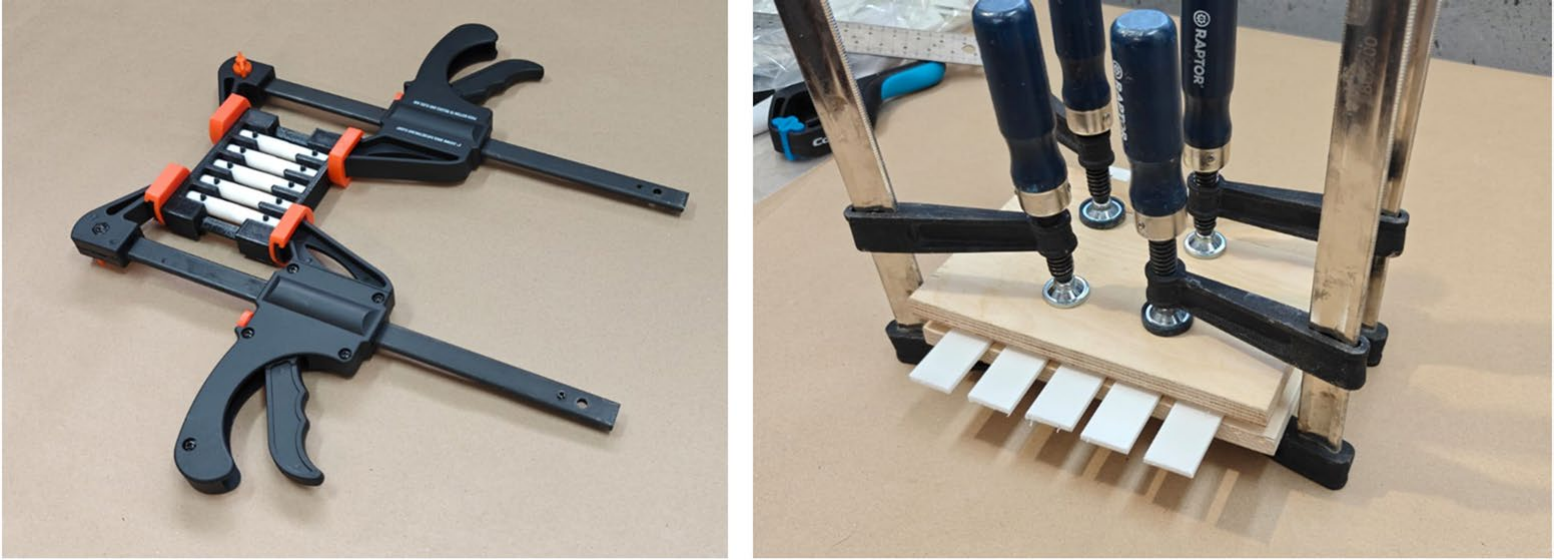
Jig for optimal adhesion of tensile, peel and shear (jig on the right side was used for shear and peel) testing specimens.

Figure 1 offers photos of the plates and jigs used to prepare specimens for the shear, peel, and tensile testing.

Application of each adhesive was done as per manufacturer guidelines. Clamping pressure for optimal adhesion was not specified for any of the adhesives by the manufacturer. Screw-type F-clamps were tightened by three full clockwise rotations from the point of first jaw contact. Quick-release spring clamps were fully engaged three times until the latch locked. Visual confirmation of continuous adhesive squeeze-out around the overlap was taken as practical indicators that comparable pressure had been applied to every specimen. No direct measurement of clamp load was recorded.

#### Conditioning specimens for test

After bonding, all the specimens were conditioned in a humidity chamber at 23 °C (± 2 °C) and 50% (± 5%) relative humidity. These parameters were chosen based on EN 291:2008^17^ to provide a stable, repeatable baseline. The five days also exceed the minimum recommended curing time specified by the manufacturer for each adhesive. Each sample was visually examined to verify good adhesion with no obvious faults.

#### Shear testing process

**Fig. 2:**
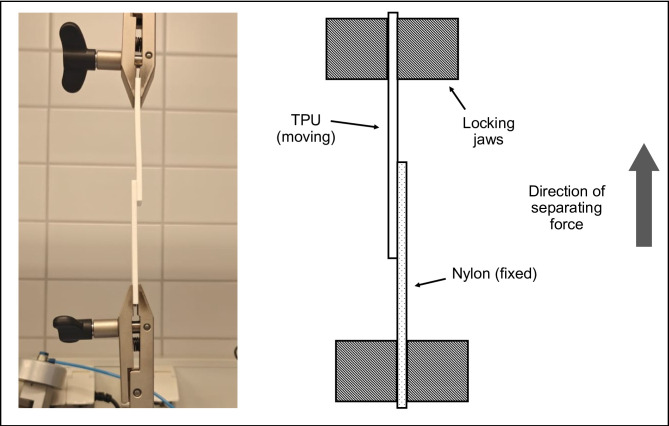
Testing setup used for shear testing with PA12 and TPU specimens in place.

The shear testing process began by installing the test specimens into the grips of the UTM, ensuring that the applied load aligned with the long axis of each specimen. The autographic mechanism and chart were adjusted to zero before starting the UTM and the load was applied at a controlled crosshead speed 10 mm/min until failure. The maximum load at failure was recorded, along with the type of failure, distinguishing between cohesive and adhesive failure. Failure stress was then calculated and expressed in N/mm^2^ (megapascals) providing a standardized measure of the adhesive’s shear strength. All the shear tests were carried out in a laboratory environment maintained at 23 °C and 50% relative humidity. Figure 2 illustrates the shear testing setup.

#### Tensile testing process

**Fig. 3:**
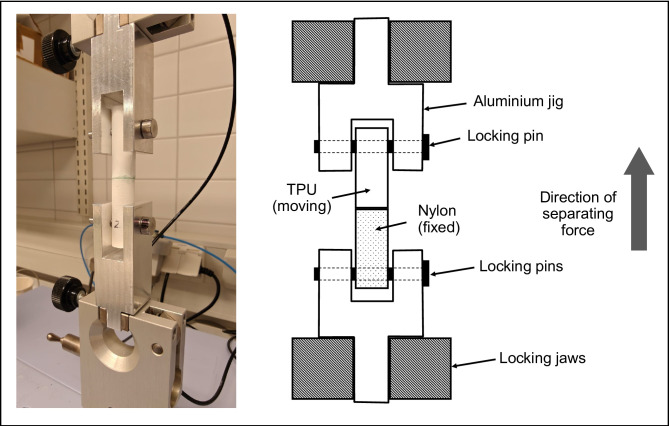
Testing setup used for tensile testing with PA12 and TPU specimens in place.

For tensile testing, specimens were installed into the UTM using locking pins and aluminum jigs, which ensured proper alignment (refer to Fig. 3). The autographic mechanism and chart were adjusted to zero before starting the UTM, and load was applied at a controlled stress rate of 22.8 MPa/min^13^ based on the surface area of adhesive bonding.

The test continued until failure, at which point the maximum load carried by the specimen was recorded. Additionally, failure modes - including cohesive failure and adherend failure, were estimated visually and documented. To determine tensile strength, the breaking load was divided by the bonded surface area, and the result was reported in N/mm^2^. All the tensile tests were carried out in a laboratory environment maintained at 23 °C and 50% relative humidity.

Figure 3 illustrates the setup for tensile testing.

#### Peel testing process

The peel or stripping test was conducted as soon as possible after removing the test specimens from the conditioning environment. It began by manually separating the free end of the 25-mm-wide flexible member from the other adherend by about 25 mm. The specimens were then installed into the UTM by clamping the free end of the rigid member in one grip while turning back and securing the free end of the flexible member in the other grip. The specimens were carefully aligned symmetrically to ensure uniform distribution of tension.

During the test, the specimen was held in the vertical plane of the clamps using an alignment plate. The power-actuated clamp securely gripped the flexible member, and the autographic mechanism and chart were adjusted to zero before starting the UTM. The flexible member was stripped from the specimen at an angle of approximately 180°, continuing until at least half of the bonded area had been separated to determine the peel or stripping strength. The result was expressed in N/mm for separation at a standard rate of 152.4 mm/min^12^. All the peel tests were carried out in a laboratory environment maintained at 23 °C and 50% relative humidity.

**Fig. 4:**
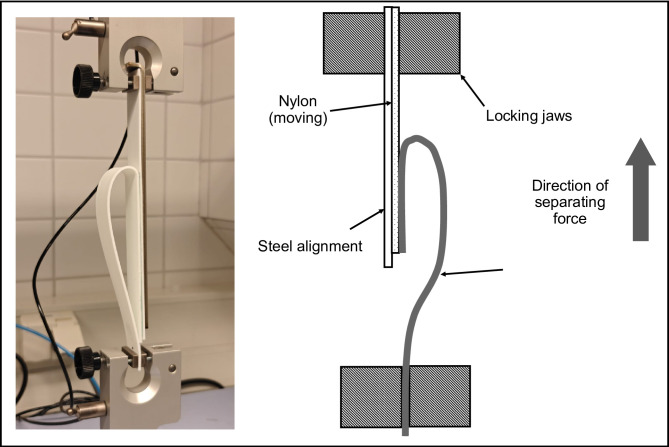
Testing setup used for peel testing with PA12 and TPU specimens in place.

Figure 4 illustrates the setup for peel testing.

#### Adaptation of the testing methods to capabilities

The standard for shear testing was adjusted to accommodate the nature of the two plastics being tested. The standards for tensile and peel testing were used as is. ASTM D3164 is designed to test lap shear between metal-adhesive-metal contacts. Using it to test PA12-adhesive-TPU is more difficult because one of the adherends is elastic and flexible. The TPU will stretch prior to the failure of the adhesive. Therefore, to reduce the time of separation to a practical value (from 30 min per sample to 5 min per sample) a jaw-separation rate of 10 mm/min was used and justified instead of the standard prescribed 1.27 mm/min.

#### Failure mode identification

Immediately after each test, the fracture faces of both specimens were visually examined under consistent bench lighting (> 1000 lx). The dominant failure mode was recorded as cohesive, adhesive, or adherend. No Scanning Electron Microscope (SEM) or optical microscopy was used in this study. For each specimen, the dominant failure mode was assigned when > 50% of the visible fracture area exhibited that mode. The same trained observer applied these criteria across all tests to ensure consistency. Inter-rater reliability agreement was not assessed in this study.

## Test results

This section presents the adhesive test results. The band graphs of the testing data include numerical values such as strength and Coefficient of Variation (COV) to help interpret the experiments better and clearly reveal the relative performance of each adhesive system. Equation (1) defines *COV*.

1 $$COV=\frac{\sigma }{\mu }*100,$$

Where, *σ* is standard deviation, and *µ* is the mean.

**Fig. 5:**
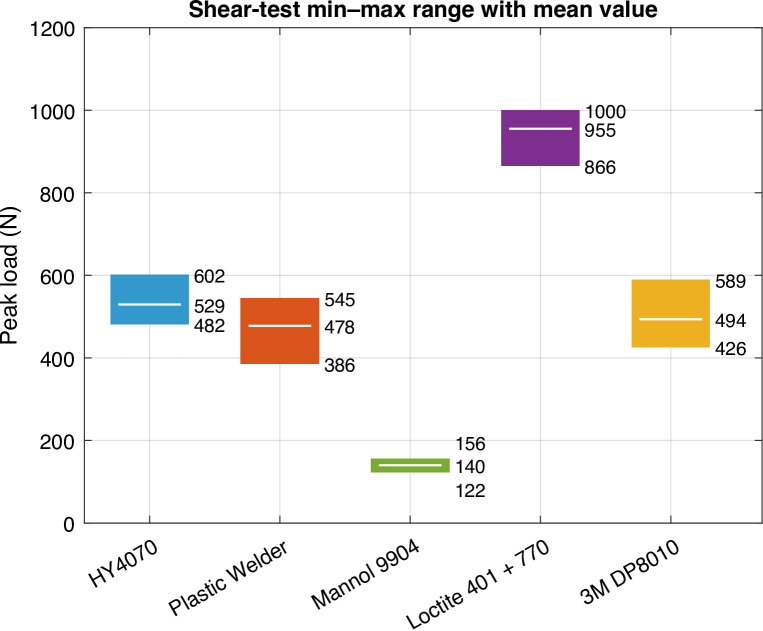
Band charts for shear testing.

The following Fig. 5 shows the bands for the shear testing. Table 1 summarizes the statistical values. Furthermore, statistics on cohesive failure (the fracture runs through the adhesive, leaving a film on both parts) and adherend failure (failure of substrate prior to adhesive) are also shown to further help clarify the results. Failure mode was assessed by visual observation of the fracture surfaces. The results are discussed in more detail in section “Discussion of test results”.

**Fig. 6:**
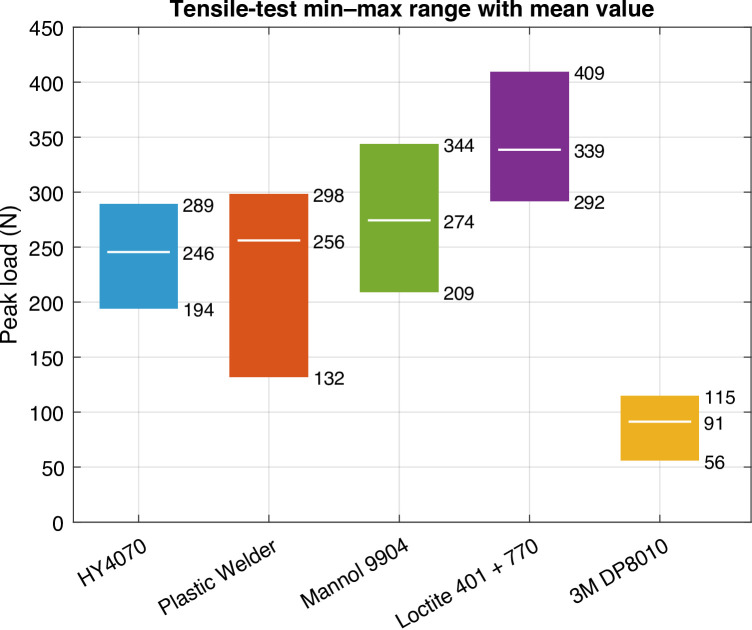
Band charts for tensile testing.

Figure 6 shows the bands for the tensile testing. Table 2 summarizes the statistical values.

**Fig. 7:**
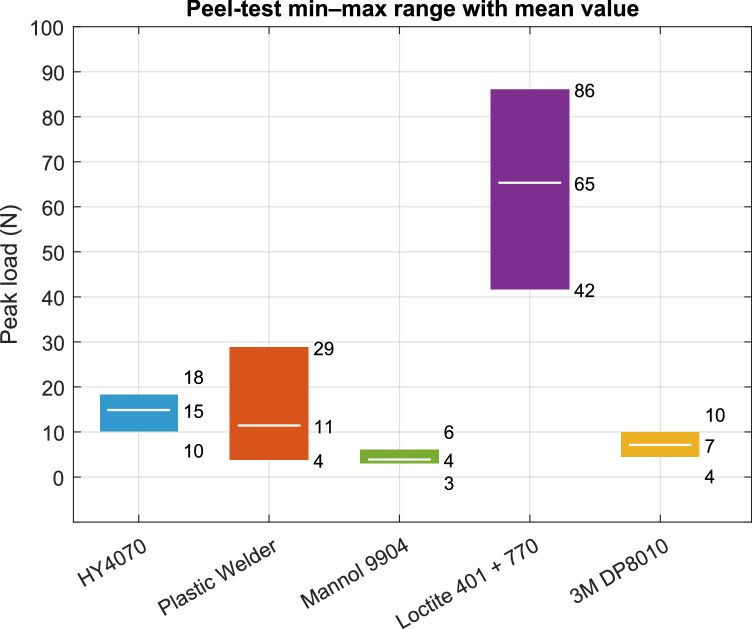
Band charts for peel testing.

Figure 7 shows the bands for the peel testing. Table 3 summarizes the statistical values.

## Discussion of test results

All three tests (shear, tensile, and peel) revealed that the adhesive bond usually broke within the adhesive and not at the surface. This suggests that adhesion to the extruded TPU and sintered PA12 was generally good for all adhesives and that bond strength was related to the material strength of the adhesive material.

### Peel failure

Peel was the most demanding test. Peak forces ranged from just 3 N up to 86 N. The Loctite 401 + 770 achieved the highest mean peel strength (2.6 N/mm) with moderate scatter. This is consistent with a thin, stiff bond-line that limits peel-arm rotation and benefits from primer-assisted wetting of PA12 and TPU. HY4070 and Permatex Plastic Welder delivered modest peel strength (0.5 N/mm), which reflected thicker and tougher bond-lines that are advantageous for impact/gap-filling but less efficient under steady peel. Mannol 9904 (epoxy) and 3M DP8010 were lowest in peel strength (0.2–0.3 N/mm). The fracture surfaces were predominantly cohesive for the top performer. Mixed cohesive/adhesive features were more frequent for the lower-performing systems. Peel strength could be improved by adding grooves to improve the surface area and including spacers between the two surfaces to precisely control the thickness of the glue line.

**Table 1:** Strength statistics for shear testing.

Property	Adhesive				
	HY4070	Plastic welder	Mannol 9904	Loctite 401 + 770	3M DP8010
Strength **(MPa)**	1.7	1.5	0.4	3.0	1.6
COV	9%	12%	9%	7%	13%
# Cohesive failures	5	5	5	3	5
# Adherend failures	0	0	0	2	0

**Table 2:** Strength statistics for tensile testing.

Property	Adhesive				
	HY4070	Plastic welder	Mannol 9904	Loctite 401 + 770	3M DP8010
Strength **(MPa)**	1.9	2.0	2.2	2.7	0.7
COV	14%	24%	17%	15%	29%
# Cohesive failures	5	5	5	5	5
# Adherend failures	0	0	0	0	0

**Table 3:** Strength statistics for peel testing.

Property	Adhesive				
	HY4070	Plastic welder	Mannol 9904	Loctite 401 + 770	3M DP8010
Strength (N/mm)	0.5	0.5	0.2	2.6	0.3
COV	30%	89%	29%	24%	29%
# Cohesive failures	5	5	5	5	5
# Adherend failures	0	0	0	0	0

### Tensile failure

Under axial tension the stress state is dominated by normal stresses. The Loctite 401 + 770 system again performed best (2.7 MPa), followed by Permatex (2.0 MPa), and HY4070 (1.9 MPa). Mannol 9904 (epoxy) achieved 2.2 MPa, indicating that the epoxy performs comparably in pure tension, even without surface pretreatment. However, performance deteriorated in shear and peel, suggesting sensitivity to interface preparation and bond-line geometry. 3M DP8010 was lowest (~ 0.7 MPa). Failures were largely cohesive except for occasional mixed modes in the lower-strength systems.

### Shear failure

In shear, the Loctite 401 + 770 system reached the highest mean (3.0 MPa) with two adherend-side failures. This indicated joint strengths approaching that of the substrate in shear. HY4070 (1.7 MPa) and Permatex (1.5 MPa) performed acceptably, with moderate scatter consistent with tougher, thicker bond-lines. 3M DP8010 ( 1.6 MPa) also performed acceptably in shear, reflecting that shear can be less sensitive to bond-line thickness for some chemistries. Mannol 9904 performed worst (0.4 MPa). Across adhesives, fractures were predominantly cohesive, with mixed cohesive/adhesive regions most visible in the lower-strength joints.

### Benchmarking of adhesives based on tests

The consistent top ranking of the Loctite 401 + 770 system suggests that primer-based surface conditioning effectively raises surface energy for both PA12 and TPU, and a thin, stiff bond-line is advantageous when peel and shear dominate. Hybrid CA/acrylic and MMA systems provide handling and toughness benefits (gap-filling, working time) but, under our no-pretreatment condition and test geometries, they cannot achieve the peak strengths of the Loctite 401 + 770 system. The epoxy’s mixed showing underscores its sensitivity to interface quality without activation. These interpretations are consistent with our observed cohesive-dominant failures.

While predominantly cohesive fracture is consistent with good interfacial adhesion under our preparation, it is not definitive. Stress concentrations at lap edges, non-uniform bond-line thickness, fillet geometry, adherend bending in peel, and minor surface irregularities can all affect the failure mode. In this study, these factors were mitigated by using fixtures, consistent clamping, and visual checks for full wet-out and squeeze-out.

**Fig. 8:**
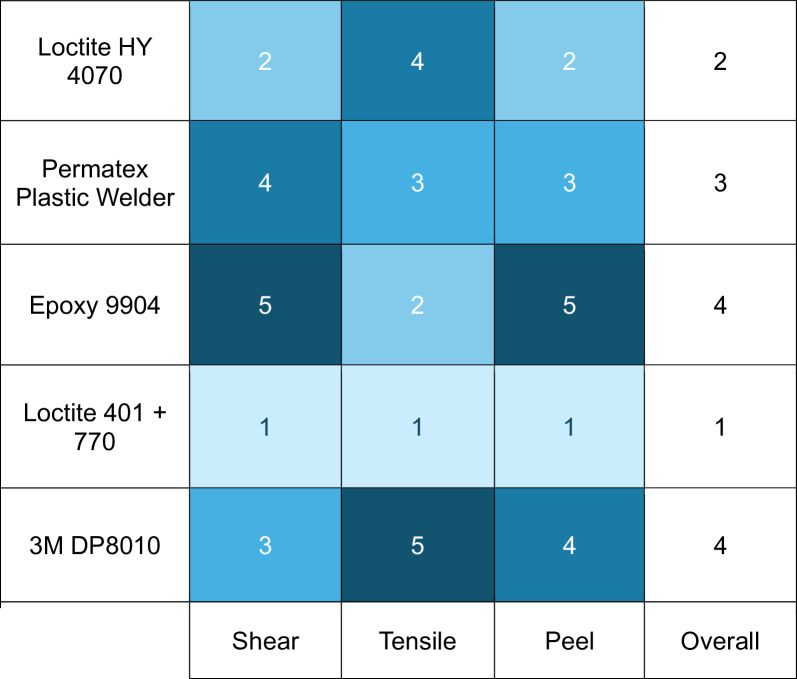
Relative rank of adhesives by load type (1 = best, 5 = worst).

Looking across all three tests, Loctite 401 used with the 770 primer is the stand-out all-rounder. Loctite HY4070 is a solid runner-up when gap-filling is needed or extra impact toughness. Figure 8 below shows a two-dimensional comparison and ranking of the adhesives across various tests.

### Adhesives ranked in decreasing ease of application

1. Loctite HY4070 – Once the mixing nozzle and plunger are installed, the adhesive is squeezed onto the adherend and bonded.
2. 3M DP8010 – This adhesive requires installation of the mixing nozzle, plunger, and dispensing gun prior to application. It can then be dispensed precisely to the adherend and bonded.
3. Loctite 401 + 770 – The primer must be applied prior to adhesion. The primer is colorless. Consistent application is necessary.
4. Mannol 9904 and Permatex Plastic Welder – Both adhesives require a spatula and a non-reactive surface on which to mix the two components. Also, it’s essential to dispense equal volumes of both components to ensure optimal strength.

### Adhesives ranked in increasing cost per unit of usage

1. Mannol 9904 and Permatex Plastic Welder – These are inexpensive adhesives that can be purchased from a store.
2. Loctite HY4070 – This is a specialist adhesive that generally must be ordered online. Mixing nozzles and plunger are included. HY4070’s smaller 11 g syringe ships with a disposable nozzle and can be hand dispensed. DP8010 needs a full-size gun.
3. Loctite 401 + 770 – The cost of Loctite 401 is comparable to Mannol 9904 and Permatex, however the 770 primer is more expensive.
4. 3M DP8010 – In addition to the adhesive cartridge, the nozzles, plunger and dispensing gun must be purchased separately. The 770 primer is pricey, but DP8010’s dedicated gun/nozzles make its initial setup cost higher.

### Limitations and future work

The number of specimens used for testing was relatively low. Five specimens were sufficient to establish trends but resulted in wide statistical confidence bands. A larger specimen size (≥ 10 per adhesive per test mode) would narrow 95% confidence intervals and let you run meaningful significance tests or Weibull analyses.

Bond-line preparation and control were hand-operated. Even tiny variations in surface cleaning, primer spread, mix ratio, or squeeze-out thickness can shift peel forces by tens of percent. Future work should use calibrated grit-blast and precision shims or glass-bead spacers to establish precise thickness. Photographing each fracture face and scoring adhesive vs. cohesive failure will also help to trace graph scatter back to process steps rather than chemistry.

Macroscopic inspection was used here for failure mode identification. Future work will include optical/SEM fractography to refine failure-mode classification and capture interfacial features not discernable by eye.

The current pool of adhesives tested does not represent all major manufacturers or all available adhesives. By improving the pool to at least 10 adhesives (2 adhesives of each type, i.e., 2 epoxies, 2 cyanoacrylates, etc.) more depth would be provided. Furthermore, additional classes of adhesive such as silicones and polyurethanes can be introduced to see their effectiveness in for this application. Another possibility would be to investigate the effect of the adhesive and adhesive + primer combinations to evaluate the effect primers have on adhesion. Testing multiple adhesives per type would also reduce the risk of outlier bias, provide more statistically robust results, and allow class-level conclusions rather than brand-specific ones.

Experimentation with improved surface treatment techniques such as plasma treatment or corona treatments is said to improve the surface wettability of low energy surfaces^18^. These treatments were not applied in this experiment mainly due to the lack of specialized equipment. While this experiment focused on the raw surface of each polymer abraded with fine sanding, the aforesaid treatments might be worth evaluating.

In summary, the testing could be improved by increasing specimen size, improved surface treatment, expanding the pool of adhesives, and tightening adhesion process controls. Those steps will turn a promising set of screening results into a more robust dataset of the five adhesives.

### Practical implications

The information provided by this experiment can be used in a prototype manufacturing context where components made from PA12 (for strength and rigidity) require an ‘overlay’ with TPU (soft and flexible) for force damping, added grip, or aesthetic value in industrial design. Such overlay applications are generally more dominant towards shear and peel type failure. Typical examples include sports equipment such as a prototype ski pole handle made of PA12 body overlaid with a TPU grip, or a robotic gripper where the gripping elements are PA12 and the gripping interface is a soft TPU.

The numerical data generated should first be regarded as a screening tool that rapidly separates clearly inadequate adhesives from promising candidates. Comparing the mean strengths clearly reveals that Mannol 9904 and 3M DP8010 were placed last in the overall scoring. Loctite 401 + 770, Permatex plastic welder, and HY 4070 consistently delivered the highest loads with comparatively low scatter. Consequently, only the latter group should be carried forward into subsequent prototyping unless a rejected product offers some unique, non-structural benefit such as color, flexibility, or unusually low cost.

The coefficients of variation reveal how sensitive each adhesive is to workmanship. Products exhibiting COV values close to 15%, most notably HY4070 and Loctite 401 + 770 are comparatively tolerant of minor process fluctuations. Conversely, adhesives with COVs exceeding 25% demand stricter monitoring, perhaps including bond-line thickness gauges, primer-coverage verification, or clamping pressure monitoring to avoid failures caused by occasional weak joints.

Finally, this experiment should be viewed as a baseline for a more comprehensive qualification effort that includes ageing, low-temperature impact, ultraviolet exposure, and fatigue or step-load testing. Designing the next test matrix around the top three performers will concentrate resources where they matter most and validate whether the apparent leaders maintain their advantage under realistic service conditions. Sharing an anonymized summary of the findings with adhesive suppliers may prompt recommendations such as alternative primers or post-cures that further improve strength or reduce scatter, turning an already useful data set into a robust foundation for both design and manufacturing.

## Conclusions

This section presents a summary of key findings of this study.

- The top performer was Loctite 401 + 770 primer. It delivered the highest mean strengths across all tests: peel = 2.6 N/mm, lap-shear = 3.0 MPa, tensile = 2.7 MPa. Variability was low-to-moderate (COV: peel 24%, shear 7%, tensile 15%).
- The mid-tier performers were Loctite HY4070 and Permatex Plastic Welder. They were competitive in shear (1.7 and 1.5 MPa) and tensile (1.9 and 2.0 MPa), and they offer useful gap-filling/handling properties. Peel performance was modest (0.5 N/mm) with higher variability for Permatex in peel.
- The under-performers in the modes selected were Mannol 9904 and 3M DP8010. Mannol 9904 showed limited shear (0.4 MPa) and peel (0.2 N/mm), while 3M DP8010 was weak in tensile (0.7 MPa) and peel (0.3 N/mm).
- The failure modes were predominantly cohesive across tests with two adherend-side failures in shear for Loctite 401 + 770, which indicated joint strengths approaching substrate limits.
- For TPU + PA12 bonded composites, where peel/shear dominate and thin bond lines are visually/functionally acceptable, Loctite 401 + 770 offers a fast, low-equipment route. The HY4070 or Permatex Plastic Welder adhesives are more suited to thicker gaps or where higher impact toughness or more working time is needed.
- Next steps include formal rate-sensitivity checks, fixed bond line thickness (shims/beads), expanded adhesive pool, and durability qualification (aging, low-temperature impact, UV, fatigue/step-load).

## Data Availability

The datasets generated and/or analyzed during the current study are large and therefore not publicly available. They are, however, available from the corresponding author on reasonable request.
